# Eating Choices—The Roles of Motivation and Health Literacy: A Cross-Sectional Study

**DOI:** 10.3390/nu14194026

**Published:** 2022-09-28

**Authors:** Urszula Zwierczyk, Christoph Sowada, Mariusz Duplaga

**Affiliations:** 1 Department of Health Promotion and e-Health, Institute of Public Health, Faculty of Health Sciences, Jagiellonian University Medical College, Skawińska Str. 8, 31-066 Krakow, Poland; 2 Department of Health Economics and Social Security, Institute of Public Health, Faculty of Health Sciences, Jagiellonian University Medical College, Skawińska Str. 8, 31-066 Krakow, Poland

**Keywords:** food choices, eating motivations, marketing, advertising, food literacy, health literacy, e-health literacy, digital health literacy

## Abstract

Food choices are determined by intrinsic and extrinsic product characteristics, biological and physiological features, psychological factors, and situational and socio-cultural factors. Self-determination theory offers the explanation of health behavior change identifying motivations located along a continuum of autonomy. Another approach to the motivations guiding health behaviors, including food choices, relies on distinguishing thematic categories. Health motivations seem to be an obvious determinant of health behaviors, but final decisions regarding health are also the effect of other types of motivations such as economic, cultural, or emotional. The role of marketing pressure in modern society is perceived to be an important source of motivation for purchasing food and other products. The Motivation–Opportunity–Ability (MOA) framework was initially proposed in order to explain the processing of brand information from advertisements and was later expanded to other areas, including health and nutritional behaviors. The aim of this study was the analysis of determinants of food choices. We have developed a common regression model including six categories of motivations addressed by the Eating Motivations Scale and three health literacy types corresponding with element of ability from the MOA framework, adjusted for socio-demographic factors, health status, and the use of the Internet and TV. The analysis was performed on data from a computer-assisted web-based interviewing (CAWI) survey among 2008 adult Internet users completed in May 2022. The uni- and multivariate linear regression models were developed with the Index of Unhealthy Food Choices (IUFC), calculated based on the responses to items asking about the frequency of the consumption of twelve food categories. Univariate modeling revealed that IUFC is significantly associated with health, food, and e-health literacies and with five out of six eating motivations. However, the multivariate regression model yielded significant associations only for eating motivations but not for the three literacy scores. Health motivation was negatively associated with IUFC (B, standard error (SE): 0.83, 0.07; 95% confidence interval (95% CI): 0.98–0.69), but positively with emotional (B, SE: 0.22, 0.04; 95% CI: 0.14–0.3), economic (B, SE: 0.41, 0.08; 95% CI: 0.25–0.56), and marketing (B, SE: 0.62, 0.08; 95% CI: 0.47–0.78) motivations. Our findings suggest that motivations guiding food choices may prevail over the element of ‘ability’ distinguished in the frameworks and models that explain people’s behaviors, including behaviors relating to health. Thus, it is essential to emphasize development of appropriate motivations and not only to provide knowledge and skills. Furthermore, one should also remember motivations other than health motivations when searching for the determinants of health behaviors.

## 1. Introduction

Adequate nutrition and physical activity are the main components of a healthy lifestyle [1]. Inappropriate nutrition may increase the risk of many chronic conditions, especially cardiovascular and metabolic diseases and cancer [1]. Harmful eating behaviors and food choices are indicated as the most important causes of increasing prevalence of overweight and obesity in many countries [2]. Unfavorable trends associated with the consumption of highly processed food are propelled by many circumstances stemming from the modern lifestyle, including commercial pressure from the food industry [3].

Eating behavior and food choices depend on many factors, including intrinsic and extrinsic product characteristics (perception and expectation), biological and physiological factors, psychological factors, situational factors, and socio-cultural factors [4]. A product’s intrinsic characteristics are related to its perception and include such aspects as its appearance, taste, and smell, but also a feeling of irritation or boredom on the side of the individual. In turn, extrinsic product characteristics depend more on the individual’s expectations associated with features such as claims of the provider, brand, packaging, and risk perception [4]. Psychological factors depend on features such as cognition, memory, and personality traits. Biological and physiological factors include genetic factors, age and gender, and gastrointestinal physiology. Finally, situational factors are associated with social and physical surroundings, issues related to coping, and assimilation [4]. Understanding, predicting, and influencing food choice behavior require knowledge from many psychological theories related to perception, learning and memory, motivation and emotion, decision-making, cognition, and social behavior [5]. As reflected by the theories described below, among the psychological factors, motivations and abilities play especially important roles in determining eating behaviors and food choices.

Nutrition is one of the most important health-related behaviors targeted by many interventions developed in public health and health promotion. Describing the mechanism underlying health behavior change is critical for developing effective interventions leading to the improvement and maintenance of health, including eating behaviors [6]. Self-determination theory (SDT) has been indicated as one of the key theories in developing strategies to influence health behaviors. SDT was elaborated seeking to explain human motivation and understand to what degree behaviors are controlled or autonomous [7]. Autonomous motivation is the basis for self-determination in human behavior, and it includes both intrinsic and integrated extrinsic motivation. Intrinsic motivation results in performing an activity without external stimuli or rewards because it somehow responds to psychological needs for competence, relatedness, and autonomy [8]. Extrinsic motivation propels activities because a person obtains the desired outcome through them. This may entail the acceptance of other people or avoiding punishment. Processes such as internalization or integration lead to the autonomization of extrinsic motivation. These two types of motivation are enhanced in various environments which people inhabit, e.g., home, school, work. According to attribution theory, extrinsic motivation occurs when we are convinced that the causes for our behaviors (perceived locus of causality) are external to ourselves [9]. Meanwhile, intrinsic motivation prevails when we believe these causes are internal or within ourselves.

The Self-Determination Health Behavior Model explains human lifestyle as the result of the interplay of a person’s experience of autonomy, competence, and relatedness, influenced by health care support, with autonomy and the intrinsic and extrinsic character of individual aspirations [10,11]. According to this model, people feeling support for their psychological needs experience improved mental health, higher quality of life, and more favorable health outcomes, including health behaviors or adherence to therapy. SDT assumes that health behaviors are propelled by many motivations positioned at various points along a continuum of autonomy [8]. The degree to which motivation is autonomous is associated with the likelihood of obtaining long-term health behavior modification. Therefore, acting for the inherent enjoyment of the activity (intrinsic motivation), following personal goals or values (integrated regulation), or obtaining individually demanded outcomes (identified regulation) will supposedly be associated with a more stable effect of behavior change than not autonomous motivations. The latter result from external forces and may take the form of introjected motivation (as in the case of focus on approval from self and others), external regulation (focus on external rewards or punishments) and finally, amotivation associated with lack of perceived competence or lack of value [12].

According to Ng et al., SDT offers an adequate theoretical framework to explain how motivation influences health behaviors [13]. Meta-analysis confirms a positive relationship between psychological need satisfaction and autonomous motivation, and beneficial health outcomes [13]. Additionally, in 2012, Verstuyf et al. reported that STD might be a promising framework for understanding the motivational process in eating regulation [14]. The association between external motivation and negative changes in food choices was reported by Hartmann et al. [15]. They also found that autonomous motivation predicts improved food choices in both genders. Kadhim et al. conducted a study analyzing the relationships between six motivations originating from SDT and unhealthy eating [16]. They observed that integrated, introjected, external regulation and amotivation were associated with a higher frequency of unhealthy eating.

According to the meta-analysis of 74 intervention studies prepared by Gillison et al., the potential scale of effect of modification of health behavior depends on targeted theoretical mediators; it can be small in the case of relatedness satisfaction and autonomous motivation, moderate in the case of competence satisfaction and large in the case of autonomy support [17]. Simultaneously, the authors suggested that creating a need supporting environment should be achieved by combining multiple techniques because individual strategies have limited independent impact on outcomes [17]. A meta-analysis published recently by Ntouamanis et al. showed that the interventions based on SDT in the area of health promotion and disease management can be, at least to some degree, effective [18]. They also found that increased need support and autonomous motivation were associated with favorable modification of health behaviors.

Apart from motivation, health-related abilities have been indicated as an important determinant of health behaviors. A better understanding of the interrelationships between the determinants of individual decisions related to health led to new models which strove to combine motivation and abilities. In 1993, Moorman and Matulich proposed the Model of Consumers’ Preventive Health Behaviors, postulating the roles of health motivation and health ability, reflecting health knowledge, as critical factors for undertaking beneficial health behaviors [19]. They found that health motivation individually facilitates health behaviors, and the presence of health motivation strengthens the effect of health ability or health knowledge. The authors emphasized that they proved their initial hypothesis that health knowledge is associated with health behaviors only with the occurrence of sufficient motivation. Specifically, they showed that in the presence of high health motivation, health knowledge is positively related to media source use and diet restrictions [19]. These findings were later confirmed by other authors, in relation to nutritional behaviors, among other things. For example, Lindbloom et al. showed that individuals with high health-related motivation and higher levels of knowledge about nutrients’ dietary functions manifest more beneficial nutritional behaviors than those with low health-related motivation but a high level of knowledge [20].

The model developed by Moorman and Matulich is related to the Motivation–Opportunity–Ability Framework proposed initially for analyzing the processing of brand information from advertisements and purchasing behaviors [21]. The MOA framework was quickly extended to other domains, including health and nutritional behaviors [22,23].

The research on the determinants of food selection and purchasing addressed many possible variables: sociodemographic and economic factors, cultural and geographic differences, religious circumstances, and, finally, psychological concepts, including attitudes and motivations. Motivations are probably one of the most frequently researched determinants among psychological factors. There are several instruments measuring eating motivations and related concepts [24], including the Eating Motivation Survey (TEMS) [25], the Eating Motivation Scale (EATMOT) [26], the Food Choice Motives Questionnaire [27], the Food Choice Questionnaire [28], and the Measure of Food Choice Values [29]. Some of the tools are focused on the specific context, e.g., ethical motivations (Measurement of Ethical Food Motives) [30], psychological motivations to eat or to abstain, including coping, social compliance, and pleasure motivations (The Motivations to Eat Measure) [31], hedonic eating behaviors (Palatable Eating Motives Scale) [32], orientation towards health and hedonic characteristics food on the market (Health and Taste Attitudes Scales) [33].

EATMOT has been proposed as a tool that can be used for various objectives in diversified populations and geographical locations [24]. As it seems, multiple subpopulations and communities may differ significantly regarding the motivations playing a key role in food selection. EATMOT may be used to adjust health communication addressed to them concerning recommended nutritional behaviors. The EATMOT instrument does not directly refer to specific motivation theories; instead, it focuses on assessing various dimensions of the motivation behind eating behaviors and food choices. These six dimensions include health, emotional, economic and availability, social and cultural, environmental and political, and marketing and advertisement motivations [26].

The knowledge and skills required for handling health information have been conceptualized as health literacy (HL). In the Outcome Model of Health promotion interventions, HL measures the effectiveness of health education and communication [34]. However, it is obvious that earlier interactions with health care systems and health professionals also influence the HL level. According to the World Health Organization, HL is defined as “the cognitive and social skills which determine the motivation and ability of individuals to gain access, understand and use information in ways which promote and maintain good health” [35].

During the 9th Global Conference of Health Promotion held in Shanghai in 2016, HL was announced as a critical determinant of health [36]. The COVID-19 pandemic augmented the interest in health and e-health literacies, as they were seen to be factors that increased adherence to preventive measures and protection against misinformation [37,38,39,40]. Available evidence suggests that HL exerts an impact on many aspects of people’s behaviors relevant to health promotion, disease prevention, and health care [41,42]; this is also true in the context of the COVID-19 pandemic [43].

It should also be noted that many specialized types of health-related literacies have been proposed and relevant instruments developed. Nutrition is such an area. Currently, there are many tools used for the assessment of food (FL) [44] and nutrition literacy (NL) [45,46]. Understanding these food and nutrition literacy concepts is extended to various aspects of handling information and decisions related to food selection, purchasing, meal preparation, and eating habits [47,48].

The number of publications measuring the levels of various types of HL and their impact on health behaviors and interactions with health care systems is very extensive [49,50]. However, the effect of health literacies on health behaviors has relatively rarely been assessed in common models with psychological constructs, including motivations. We believe it would be interesting to determine the roles of health literacies, including FL and various dimensions of motivation in guiding eating choices after adjusting for sociodemographic and economic factors, health status, and information sources.

This study’s main aim was to analyze the factors determining the food choices of adult Internet users in Poland. It is usually assumed that HL and other health-related types of literacy, e.g., FL or e-health literacy (eHL), exert a positive effect on health-related behaviors. However, the roles of these literacies have rarely been analyzed in common models of health behaviors along with the motivations of respondents. As shown earlier, key theories explaining individual behaviors, including health behaviors, address the role of motivations. In SDT, motivation is the main construct explaining the drivers of human behaviors. In the MOA model, motivation is combined with opportunities and abilities. Therefore, we have decided to develop a multivariate model of eating choices encompassing three types of health-related literacies and scores based on subscales of the EATMOT tool, adjusting for sociodemographic variables, health status, and the use of Internet and TV in the adult population. We have also adjusted the effects of motivations and health literacies for the size of the household as it can be related to unhealthy eating patterns [51,52]. In the cluster of independent variables used in regression modeling we also included the prevalence of chronic disease and self-assessment of health status to adjust for potential adherence to healthy eating related to medical circumstances. We have also assessed the role of motivations other than only health-related ones in determining food choices. We assumed the following main hypotheses related to the roles of motivations and health literacies:

- Eating motivations are significantly associated with food choices with health, environmental, social, and cultural motivations showing a negative relationship with harmful food choices and economic, political, marketing influenced motivations showing a positive relationship;
- Health literacy, digital health literacy, and food literacy are significantly negatively associated with harmful food choices;
- The effect of motivations and literacies is maintained in an adjusted model of food choices.

The model was applied to the data from a survey of a large sample representative of Internet users in Poland. The selection of this sample was based the fact that the number of regular Internet users is steadily growing in Poland, and the fraction of the population not using the Internet is mainly limited to the oldest strata. We were also interested in the influence of the Internet and digital media on health behaviors. Finally, we wanted to analyze the relationship between food choices and digital health literacy.

## 2. Materials and Methods

### 2.1. Survey

The analysis reported in this paper is based on the data from an online (computer-assisted web-interviewing, CAWI) survey performed on a representative sample of 2008 Internet users in Poland in May 2022. The survey was conducted by the PBS Company [53], specialized in opinion and market research, selected as the result of the bidding procedure for offers that is obligatory for public organizations. Given the size of the target population of 28,600,000 [54], a fraction of 0.5 and a confidence level of 0.95, the sampling error based on the size of the study sample is about 2.2%. The structure of the study sample was adjusted to conform to the structure of the population of adult Internet users in Poland in reference to age, gender, level of education, place of residence, and territorial unit (voivodship) [54]. The final number of 2008 questionnaires was obtained after excluding 769 questionnaires that did not comply with the quality criteria (unfulfilled recruitment criteria, refusal to participating in the survey after starting the survey, inappropriate response to control questions, too short response time, and tendency in responses).

The study was accepted by the Bioethical Committee of the Jagiellonian University in Krakow, Poland (Consent No 1072.6120.197.2021 from 29 September 2021, with amendments). The respondents were invited to join the study from the Internet panel ‘Poznaj.to’ [55] maintained by the PBS Company [53]. After receiving the information about the survey’s aims, potential participants were asked to provide informed consent before filling out the online questionnaire. Respondents could resign from participating in the study at any moment.

### 2.2. Questionnaire

The questionnaire included 86 individual items. It encompassed the 23-item Eating Motivation Scale (EATMOT) [56], the 10-item e-Health Literacy Scale (eHEALS) [57,58], the 11-item Short Food Literacy Questionnaire (SFLQ), the 6-item European Health Literacy Survey Questionnaire (HLS-EU-Q6) [59,60], a set of items asking about the frequency of the consumption of selected foods and items asking about other health behaviors. There were also three questions about the frequency of accessing lifestyle influencers’ websites, daily Internet usage, and watching TV. Finally, the questionnaire included a set of questions about sociodemographic and economic characteristics.

### 2.3. Measures

#### 2.3.1. Index of Unhealth Food Choices (IUFC)

The score reflecting food choices that are potentially unhealthy (Index of Unhealthy Food Choices, IUFC) was calculated based on the responses to twelve items asking about the frequency of the consumption of selected types of food. The list of the kinds of food was established arbitrarily but it did follow other tools recommended and used for assessing nutritional behaviors in Poland [61]. The main assumption behind this list was the selection of the representative types of food in terms of possible harmful or beneficial effects on health. The Authors are aware of a certain simplification of such an approach; however, the detailed assessment of the nutritional habits of the respondents was not a primary goal of this study. The responses to items could be provided according to a frequency scale ranging from ‘I do not eat/drink such food/beverages’ to ‘every day’. Values from 1 to 7 were assigned to response options. The scoring was reversed when the items asked about the frequency of consuming whole meal bread, fish, fruit and vegetables. The total score achieved higher values in the case of more frequent consumption of foods with unfavorable impact on health, e.g., meat, and less frequent consumption of recommended food, e.g., fruit and vegetables. The total score could range from 12 for the most beneficial to 84 for the most harmful eating habits. The internal consistency of this ad hoc scale was not fully satisfactory as the Cronbach-α coefficient was below 0.7 (0.642), and the Guttman split-half coefficient was only 0.507.

#### 2.3.2. Eating Motivation Scale (EATMOT)

The EATMOT scale was developed by Ferrão et al. [62] and later validated by an international team of researchers [26,56]. The version used in this study consisted of 23 items assessing six types of motivations: health (4 items), emotional (5 items), economic and availability (3 items), social and cultural (3 items), environmental and political (5 items) and related to marketing and advertisements (3 items). The responses to the items included in the instrument are provided according to the 5-item Likert scale, from ‘strongly disagree’ to ‘strongly agree’, with a neutral response in the middle. Responses on the Likert scale were converted to numerical values from 1 to 5. For every subscale, the score expressing the level of relevant motivation type was calculated as a sum of the individual scores of the included items. The values of the Cronbach-α and Guttman split-half coefficients for the EATMOT subscales were 0.806 and 0.790 for health motivation, 0.849 and 0.856 for emotional motivation, 0.619 and 0.506 for economic and availability motivation, 0.447 and 0.391 for social and cultural motivation, 0.798 and 0.744 for environmental and political motivation, and finally 0.724 and 0.644 for motivation related to marketing and advertising. These values show that internal consistency was insufficient in the case of the subscale reflecting economic and availability motivation and social and cultural motivation.

#### 2.3.3. Six-Item European Health Literacy Survey Questionnaire (HLS-EU-Q6)

HLS-EU-Q6 is a short version of the questionnaire developed within the European Health Literacy Survey project [59,60]. Respondents respond to items in the questionnaire based on options reflecting the difficulty level of accomplishing tasks measuring various aspects and contexts of health literacy. The response ‘very difficult’ is assigned value 1, ‘fairly difficult’ value 2, ‘easy’ value 3, and ‘very easy’ value 4. The response option ‘difficult to say/not applicable’ is a missing value. The score is calculated as a mean of individual scores if there is not more than one missing value. The score ≤ 2 is interpreted as inadequate, from >2 to 3 as problematic, and >3 as sufficient for HL. To avoid excluding a significant number of observations from regression modeling due to missing values for the HL score, the category of ‘undetermined HL’ was distinguished. The coefficient of reliability, the Cronbach-α coefficient, and the Guttman split-half coefficient were 0.848 and 0.820, respectively, indicating good internal consistency.

#### 2.3.4. Short Food Literacy Questionnaire (SFLQ)

The SFLQ, initially developed by Krause et al., consists of 12 items. In the version adapted to Polish, after exploratory factor analysis, 11 items were retained [63]. The responses to individual items are selected from scales encompassing 4–6 options. The final score is calculated as a sum of individual scores after converting response options to numerical values as described by Krause et al. [44]. The score can assume values from 6 to 48. Internal consistency assessed with the Cronbach-α coefficient was good as it was equal to 0.833. The Guttman split-half coefficient was 0.718.

#### 2.3.5. e-Health Literacy Scale (eHEALS)

The eHEALS is a popular tool used for assessing digital health literacy, introduced by Norman and Skinner in 2006. The tool was adapted to Polish on large samples included in telephone- and Internet-based surveys [58]. The eHL score is calculated based on the responses given to 8 items based on the 5-item Likert scale from ‘decidedly disagree’ to ‘decidedly agree’. The response options are converted to numerical values from 1 to 5. The final score can assume values from 8 to 40. The Cronbach-α coefficient was 0.903 and the Guttman split-half coefficient 0.854.

### 2.4. Statistical Analysis

The statistical analysis was conducted with the IBM SPSS Statistics v.28 package (IBM Corp., Armonk, NY, USA). Descriptive statistics were calculated for the variables included in the analysis; categorical variables were described with absolute and relative frequencies. The mean and standard deviation (SD) were provided for continuous numerical variables.

The predictors of IUFC were assessed with univariate and multivariate linear regression models. The IUFC score was calculated based on the responses to twelve items asking about the frequency of the consumption of selected types of food and beverages. Independent variables used in the regression models included: HL, FL, eHL, scores reflecting eating motivations, age, sociodemographic and economic variables (age, gender, place of residence, education, vocational status, marital status, monthly income per household member), the involvement in food purchases, number of people in a household, self-assessment of health status and the prevalence of chronic disease, accessing influencers’ websites, and daily duration of Internet use and TV watching. The categorical sociodemographic variables included in the analysis could assume the following response options: gender—female or male, place of residence—six response options from ‘rural’ to ‘urban above 500,000 inhabitants’, education—from ‘lower than secondary’ to ‘university—Masters’, marital status—4 options (‘married’, ‘in partnership’, ‘single’, and ‘widowed, divorced or separated’), vocational status—6 options (‘employee’, ‘self-employed or farmer’, ‘retired or on a disability pension’, ‘high school or university student’, ‘vocationally passive incl. unemployed’), monthly net income per household member—5 options from ‘1501–3000 PLN’ to ‘above 5000 PLN’, and ‘refusal to reveal’, number of people in a household—5 options from ‘1 person’ to ‘more than 4 persons’. The response options for the variable reflecting involvement in purchases of consumed food could be provided according to a frequency scale from ‘sometimes or less often’ to ‘always’. The prevalence of chronic diseases was a dichotomous variable (No/Yes), and self-assessment of health status was based on a 5-point scale from ‘unsatisfactory’ to ‘perfect’. Finally, the access to influencers’ websites could be answered with five options, from ‘no use’ to ‘at least 4 times a week’, daily use of the Internet with the scale from ‘not more than 1 h’ to ‘>5 h’ and daily TV watching with the scale from ‘no use’ to ‘>4 h’.

Unstandardized regression coefficients (b), standard errors (SE), standardized regression coefficients (β), 95% confidence intervals (95% CI), and *p*-values were provided for independent variables included in uni- and multivariate linear regression models. The ANOVA test was performed for regression models, and R2 was calculated. Ascertaining the lack of correlation of the random components was assessed with the Durbin–Watson test. The multicollinearity of variables included in the multivariate model was evaluated based on the variance inflation factor (VIP) and tolerance. *p*-values < 0.05 were assumed significant.

## 3. Results

### 3.1. Characteristics of the Study Group

The mean age (SD) of the respondents was 40.00 (12.80) years. In the sample of 2008 respondents, 50.80% (*n* = 1020) were women, 37.65% (*n* = 756) were residents of rural areas and 11.70% (*n* = 235) were residents of urban areas with >500,000 inhabitants, and 34.81% (*n* = 699) were persons with university education. The percentage of employees was 52.04% (*n* = 1045), self-employed or farmers 12.85% (*n* = 258), and retired or on disability pension 8.22% (*n* = 165). Detailed characteristics of the study group are shown in Table 1.

HL remained undetermined for 21.1% (*n* = 423) of respondents. Its mean level (SD) was 24.09 (7.25). In the study group, inadequate HL was found in 10.3% (*n* = 206), problematic in 59.4% (*n* = 1193), and sufficient in 9.3% (*n* = 186). The mean eHL (SD) was 29.42 (5.06), and the mean FL—was 31.40 (6.64).

### 3.2. Food Choices

The distribution of the responses to items asking about the frequency of the consumption of selected types of food and beverages and individual scores derived from these items are shown in Table 2. Individual scores for items asking about food and beverages with potentially unfavorable health effects spanned from 2.82 (SD = 1.30) for fast food to 5.27 (SD = 1.24) for meat. In the case of whole meal bread, the individual score was 3.53 (SD = 1.72), for fish it was 4.62 (1.46), and for fruit and vegetables it was 2.92 (1.48). The mean IUFC (SD) was 42.40 (8.39).

### 3.3. Univariate Linear Regression

Univariate models revealed that the IUFC was significantly associated with HL, eHL, FL, and scores reflecting health-related, emotional, economic, environmental, and marketing and advertising motivations (Table 3). The IUFC was higher among respondents with inadequate HL than those with problematic HL (B, SE: 1.62, 0.63; 95% CI: 0.38–2.86). No significant differences existed between persons with sufficient or undetermined HL and problematic HL. The IUFC was lower among respondents with higher eHL (B, SE: −0.11, 0.04, 95% CI: −0.18–−0.04) and with higher FL (B, SE: −0.16, 0.03; 95% CI: −0.21–−0.11). The persons with higher health-related (B, SE: −0.96, 0.07, 95% CI: −1.09–−0.83) and environmental (B, SE: −0.46, 0.05; 95% CI: −0.57–−0.36) eating motivations had a lower IUFC. Respondents with higher emotional or economic eating motivations, or eating motivations related to marketing and advertising showed a higher IUFC.

Among sociodemographic and economic variables, only place of residence and income levels were not significantly associated with the IUFC. Older respondents showed lower IUFC than younger ones (B, SE: −0.21, 0.01; 95% CI: −0.24–−0.19). Males showed decidedly less favorable eating habits than women (B, SE: 3.38, 0.37; 95% CI: 2.66–4.10). A higher level of attained education was associated with a more favorable eating pattern (B, SE for the comparison between persons with university Masters and secondary education: −1.72, 0.50; 95% CI: −2.7–−0.73). Interestingly, widowed or divorced persons showed more favorable eating habits than married ones. In turn, persons living in partnership or singles had higher IUFC than married persons. Lower IUFC was also found among self-employed people and farmers, and among retired people or those on disability pension than in employees.

The prevalence of chronic diseases was associated with higher IUFC (B, SE: 1.41, 0.59; 95% CI, 0.24–2.57). Persons assessing their health status as satisfactory presented significantly lower IUFC than those considering it good (B, SE: −1.71, 0.51; 95% CI: −2.71–−0.72).

Persons living in households with fewer than three members showed significantly lower IUFC, and those living in homes with more than four inhabitants had significantly higher IUFC. Respondents often or very often involved in purchasing the food they consumed showed higher IUFC than those involved in purchases ‘almost always’.

Finally, respondents spending more than 4 h on the Internet had significantly higher IUFC than those using the Internet for >2 to 3 h daily (B, SE: 1.75, 0.67; 95% CI: 0.43–3.07). Less favorable nutritional patterns were also found in the case of respondents watching TV for more than 4 h daily (B, SE: 1.54, 0.71; 95% CI: 0.16–2.93), and more favorable were found among those who watched TV for less than 0.5 h daily (B, SE: −1.97, 0.73; 95% CI: −3.4–−0.55). Unexpectedly, persons accessing lifestyle influencers’ websites 2–3 times a week had significantly higher IUFC than those not accessing such websites (B, SE: 1.93, 0.62; 95% CI: 0.71–3.14).

### 3.4. Multiple Linear Regression

The multiple linear regression model was significant (ANOVA, F = 18.584, *p* < 0.001) with an R2 of 0.368 (corrected R2 of 0.348). The Durbin-Watson statistic value was 1.955, confirming no autocorrelation in the residuals from regression analysis.

The multiple models retained significant associations with IUFC for health-related, emotional, economic, and related marketing and advertising motivations, age, and gender (Table 4). Significant associations were also observed for selected comparisons between categories of marital status, the number of people in a household, involvement in purchases of consumed food, daily Internet usage, and TV watching. The respondents with higher scores reflecting health-related eating motivation had lower levels of IUFC, and those with higher emotional, economic and related to marketing motivations had higher levels of IUFC.

In the multivariate model, no significant association was found in the univariate model for HL, eHL, and FL. Still, older age was associated with more beneficial nutritional patterns (B, SE: −0.18, 0.02; 95% CI: −0.22–−0.14). Males consumed more unhealthy food (B, SE: 3.54, 0.36; 95% CI: 2.84–4.23).

Persons using the Internet for more than 5 h daily had higher IUFC than those using it for >2 to 3 h. Finally, people watching TV for less than 0.5 h daily had more favorable eating patterns than those watching it for >1 to 2 h.

## 4. Discussion

In this study, we analyzed the association between the score reflecting unhealthy food choices (FCI) based on the responses to items asking about the frequency of the consumption of twelve selected types of food and beverages and eating motivations and health literacies. Univariate models showed significant associations between eating habits and sociodemographic and economic variables, three types of literacies, scores indicating eating motivations, the prevalence of chronic disease and self-assessment of health status, and finally, accessing influencers’ websites, daily usage of the Internet and daily duration of watching TV. These findings agree with a systematic review by Caso and Vecchio that confirmed that food choices are related to many factors. Those most frequently researched include sociodemographic, situational, and psychological ones [64]. The whole array of determinants was also reported for populations with significantly different cultural backgrounds. For example, among the Chinese population, food choices were influenced by: (1) principles of traditional Chinese medicine, (2) perception of a healthy diet in culture, (3) striving for harmony in families and communities, and (4) physical, social, and environmental factors. [65]. Our findings agree with the results reported by other authors as for the determinants of healthy or unhealthy eating patterns. For example, Ferreira et al. showed that the perception of healthy eating was negatively associated with being male, having lower education, practicing less physical activity and having a higher body mass index (BMI) [66]. In a Polish study, male gender and lower level of education were predictors of unhealthy eating patterns. Physical activity and BMI were not included in our study’s model developed for eating habits.

When combined in the multivariate linear regression model, many associations described earlier for the Polish population were not retained. The multiple linear regression model explained nearly 37% of the variance of the independent variable. The most striking finding from the multiple regression model is the lack of significant association between health, food, and digital health literacies with food choices. On the other hand, most eating motivations, including health motivation, have maintained significant associations with the score reflecting eating habits. It is usually assumed that health and derived types of literacy are critical factors determining people’s health behaviors. However, it should be noted that their effect has rarely been assessed combined with psychological constructs, such as motivation. In our study, we have applied the EATMOT scale to measure six thematic categories of motivations. Interestingly, health-related motivation was significantly associated with eating habits assessed in terms of health effects and other emotional, economic, and marketing-related motivations. It should also be noted that the latter three motivations showed a positive relationship with unhealthy food choices.

As expected, univariate regression models showed that all three types of literacy were significantly associated with less frequent consumption of unhealthy food. A survey performed among college students from Taiwan revealed that functional e-health literacy was negatively related to unhealthy food intake and interactive eHL was positively related with a balanced diet [67]. Furthermore, critical eHL was positively associated with regular eating habits. The assessment of the eHL was performed with the 12-item eHealth Literacy Scale.

However, a more general view of the relationship between HL and nutrition behaviors is not unequivocal. A systematic review prepared by Carrara and Schulz identified 35 associations between HL and various nutrition behaviors [68]. Among them, only five were direct positive associations, all in the general population; 20 were insignificant, of which 15 were identified in the patients’ group. The association was negative (partially or entirely mediated) in two cases.

In our study, the effect of literacies was no longer present when these variables were combined with six types of motivations in the combined model. This observation seems to agree with earlier findings of Moorman and Matulich that motivation is a factor individually influencing health behaviors, and high health ability (which can be understood as HL) is not enough to incite beneficial health behaviors when the motivation toward healthy behavior is insufficient [19]. Our analysis revealed that emotional, economic, and marketing-related motivations result in more unhealthy eating patterns. Additionally, their effects were not counteracted by the level of health, e-health, or even food literacy. Future studies should examine the contradictory effect of motivations stemming from different areas. It is also possible that in various populations, key motivations may be different. Wongprawmas et al. reported that among Italian consumers, the strongest determinants of food choices were environmental factors and health [69].

On the other hand, Gül and Erci conducted a study on a sample of adults from Turkey, assessing determinants of eating behaviors but not the choice of food types [70]. They found that when combined in a multiple linear regression model, HL and health perception accounted only for 1.3% (R2 = 0.013) of the variance of the dependent variable. Interestingly, in their analysis, HL was a significant predictor of eating habits and perception of health was not.

Jezewska-Zychowicz and Plichta assessed the determinants of the pro-healthy (PHDI) and non-healthy diet (NHDI) indexes based on the frequencies of the consumption of 24 types of food, 12 classified as healthy and 12 as unhealthy [71]. In their study, attitudes toward food and nutrition were measured based on the responses to seven statements about the connection between food, nutrition, and health (4 items), appearance, self-esteem, and lifestyle. Similar to in our study, Jezewska-Zychowicz and Plichta observed a strong propensity of males toward an unhealthy diet. One could expect more balanced gender preferences toward unhealthy eating in their research, as it was carried out among students of food and nutrition majors. Of interest also, nutrition knowledge was significantly negatively associated with NHDI but not significantly associated with PHDI. In turn, the attitudes towards food and nutrition measured by Jezewska-Zychowicz and Plichta were significantly associated with NHDI and PHDI in the adjusted model. We may assume that attitude measures may be treated as proxies of health-related motivations in our study. So, their findings are to some degree analogical to those seen in the sample of Polish Internet users, at least concerning the motivational background.

In our study, we have not analyzed in detail the sources of information about food and nutrition. It was only covered on a general level in the SFLQ. However, it seems that this aspect can also be very interesting. For example, Roy et al. observed that the score reflecting healthy diet patterns was significantly higher among the respondents influenced by nutrition experts and family and friends than others [72]. Interestingly, scores calculated for the so-called meat dietary pattern were significantly higher among those inspired by celebrity cooks but also by family.

The analysis showed that an unfavorable eating pattern is associated with longer time spent on the Internet. This relationship may be related to higher exposure to marketing content and advertisement distributed in digital media. It is also likely that people who spend more than 5 h daily on the Internet have unfavorable lifestyles. For example, their vocational activity is associated with Internet use, and they cannot take care of healthy nutrition. If they are corporate workers, they can develop the habit of ordering takeaway fast food.

A longer time spent watching TV was also associated with unhealthy eating patterns. Similar to in the case of a long time spent on the Internet, higher exposure to TV programs may result in the internalization of the marketing content promoting fast food, industrial sugar products, ready-made cereals, sugar-sweetened beverages, energy drinks, and other unhealthy products dominating the advertising time.

Our study was mainly focused on the analysis of the relationships between food choices, motivations, and health literacies. However, one should remember that this study was conducted during the pandemic and this could, to some extent, influence food choices. We have not analyzed this aspect but many authors reported various findings about the impact of the epidemic situation and especially COVID-19 lockdown on eating behavior. For example, Herle et al. described several trajectories in eating behavior in a large sample of adults from the United Kingdom, but stated that the majority (64%) of respondents showed no change in eating behavior [68]. According to Poelman et al., unhealthier eating patterns were more frequently reported during the COVID-19 pandemic by persons with overweight and obesity than those with a normal weight [73]. A narrative review prepared by Johnson et al. showed that in the initial phase of the COVID-19 pandemic eating behaviors changed insignificantly [74]. However, increased intake of meals and snacks was observed more frequently than decreased intake among those respondents whose eating behaviors changed.

In the future, we would like to address the role of motivations related to marketing and advertisement more in-depth. It is clear that so-called commercial determinants play a significant role in shaping the health behaviors of modern societies [75]. This effect has been underestimated in recent decades, even if the struggle to limit the harmful impact of tobacco smoking began many years ago [76]. The question of how we should tackle the influence of food industries offering highly processed food is particularly valid. Another direction of our planned research is to focus on the interrelationship between health-related attitudes and literacies and pro-ecological attitudes. In this context, food choices may be particularly important in limiting the harmful effects of food production on the environmental well-being of our planet [77,78].

### Limitations

The observational study does not allow for the assessment of causal relationships. Furthermore, we could not analyze the time-dependent dynamics of the observed relationships.

One of the main limitations of this study was related to the survey technique. The analysis was conducted on data coming only from Internet users. Older persons and those with lower income are underrepresented in such a group. Therefore, extrapolating our findings to the whole adult population is impossible. On the other hand, the study sample enabled the assessment of the role of digital health literacy in shaping eating habits.

Our study was focused on the role of health and other health-related literacies and various types of motivations. However, we have not analyzed the motivations according to the classifications derived from the SDT. Instead, we have applied the tool measuring various thematic dimensions of eating motivations proposed by Guiné et al. [26]. These dimensions do not correspond to the SDT classification; each EATMOT dimension may encompass various types of motivation in the autonomy continuum [7].

The tool applied to assess eating habits was an ad hoc measure stemming from an arbitrary selection of food categories to measure the health effect of a given diet. The frequency of the consumption of all products was assessed according to one scale, ranging from abstaining from eating the type of food to everyday consumption. Decidedly, such an approach to measure the health dimension of a diet should be treated cautiously; such a frequency scale is not fully compatible with the consumption patterns of all included food and beverages.

It should also be mentioned that a final number of 2008 questionnaires adhering to quality criteria, 769 questionnaires, were excluded mainly because of not fulfilling recruitment criteria, refusal to continue the questionnaire, or inappropriate responses to control questions. Such a number of the excluded questionnaire is not particularly surprising as for the earlier survey conducted within Internet panels, especially since 61% of exclusions resulted from the attempt to join the survey by persons not deemed as the target audience.

## 5. Conclusions

The lack of a significant association between the health-related measure of food choices and HL, FL, and eHL in the multivariate model is an unexpected finding of this study. Including health literacy scores and various eating motivations showed that only the latter variables retained their significant association with nutritional behaviors. Such results suggest the need for readjustment of the overwhelming effort of the community of public health and health promotion practitioners striving to develop comprehensive educational interventions to develop knowledge and skills in order to increase the health literacy and specialized types of literacies in the community. It has become evident that knowledge and skills are not enough without forming an appropriate motivational basis for health behaviors change, at least in terms of nutrition behaviors.

## Figures and Tables

**Table 1:** Characteristics of the study group.

Variable	Categories of Variable	%	*n*
Gender	female	50.80	1020
	male	49.20	988
Place of residence	rural	37.65	756
	urban below 20,000 inhabitants	12.25	246
	urban 20,000–100,000 inhabitants	20.97	421
	urban 100,000–200,000 inhabitants	8.52	171
	urban 200,000–500,000 inhabitants	8.91	179
	urban above 500,000 inhabitants	11.70	235
Education	lower than secondary	18.53	372
	secondary	34.71	697
	post-secondary non-university	11.95	240
	university Bachelors	12.30	247
	university Masters	22.51	452
Marital status	married	45.97	923
	in partnership	15.94	320
	single	27.19	546
	widowed, divorced or separated	10.91	219
Vocational status	employee	52.04	1045
	self-employed or farmer	12.85	258
	retired or on disability pension	8.22	165
	high school or university student	8.12	163
	vocationally passive incl. unemployed	18.77	377
Monthly net income per household member	not more than 1500 PLN	15.79	317
	1501–3000 PLN	36.45	732
	3001–5000 PLN	21.12	424
	more than 5000 PLN	11.80	237
	refusal of response	14.84	298
Number of people in a household	1 person (only respondent)	8.81	177
	2 persons	22.71	456
	3 persons	28.34	569
	4 persons	24.20	486
	more than 4 persons	15.94	320
Involvement in purchases of consumed food	sometimes or less often	8.47	170
	often	8.86	178
	very often	16.33	328
	almost always	27.24	547
	always	39.09	785
Chronic disease	no	56.42	1133
	yes	43.58	875
Self-assessment of health status	unsatisfactory	6.92	139
	satisfactory	19.52	392
	good	44.32	890
	very good	24.10	484
	perfect	5.13	103
Accessing influencer’s web pages	no use	35.01	703
	1–3 times in the last month	29.63	595
	once weekly	15.14	304
	2–3 times a week	12.30	247
	at least 4 times a week	7.92	159
Use of the Internet daily	not more than 1 h	8.81	177
	>1 to 2 h	20.87	419
	>2–3 h	21.71	436
	>3 to 4 h	16.73	336
	>4 to 5 h	11.60	233
	>5 h	20.27	407
Watching TV daily	no use	13.70	275
	not more than 0.5 h	9.41	189
	>0.5 to 1 h	15.99	321
	>1 to 2 h	21.86	439
	>2 to 3 h	18.48	371
	>3 to 4 h	10.31	207
	>4 h	10.26	206

Abbreviations: PLN—Polish zloty, TV—television.

**Table 2:** The results of the assessment of the frequency of the consumption of selected food and beverages.

Type of Food or Drink	Every Day	Nearly Every Day	A Few Times Weekly	Once a Week	2–3 Times in the Last Month	Once or None in the Last Month	I Do Not Eat/Drink Such Food/Beverages	Score Mean (Standard Deviation)
whole meal bread *	12.05 (242)	17.63 (354)	27.04 (543)	14.99 (301)	11.5 (231)	10.46 (210)	6.32 (127)	3.53 (1.72)
meat	13.7 (275)	29.83 (599)	40.04 (804)	9.16 (184)	3.34 (67)	1.39 (28)	2.54 (51)	5.27 (1.24)
Fish *	1.25 (25)	2.24 (45)	10.96 (220)	36.45 (732)	22.31 (448)	21.26 (427)	5.53 (111)	4.62 (1.22)
sugar confectionery selection	11.01 (221)	19.52 (392)	30.13 (605)	20.17 (405)	11.11 (223)	5.18 (104)	2.89 (58)	4.72 (1.46)
industrial sweets	5.58 (112)	12.1 (243)	26.64 (535)	20.82 (418)	16.24 (326)	11.85 (238)	6.77 (136)	4.07 (1.58)
ready-made breakfast cereals	4.28 (86)	6.77 (136)	14.74 (296)	13.99 (281)	12.6 (253)	17.98 (361)	29.63 (595)	3.04 (1.84)
fast food	1.05 (21)	2.04 (41)	7.37 (148)	17.73 (356)	25 (502)	32.52 (653)	14.29 (287)	2.82 (1.3)
fruit and vegetables *	19.97 (401)	21.46 (431)	29.63 (595)	12.6 (253)	9.51 (191)	5.78 (116)	1.05 (21)	2.92 (1.48)
ready-made sauces	1.44 (29)	3.04 (61)	10.76 (216)	18.18 (365)	19.32 (388)	23.95 (481)	23.31 (468)	2.84 (1.5)
energy drinks	2.64 (53)	4.48 (90)	7.67 (154)	7.67 (154)	7.87 (158)	16.04 (322)	53.64 (1077)	2.24 (1.71)
sugar sweetened beverages	5.03 (101)	8.42 (169)	15.04 (302)	12.55 (252)	15.94 (320)	17.68 (355)	25.35 (509)	3.2 (1.86)
alcoholic beverages	2.29 (46)	4.33 (87)	14.69 (295)	21.46 (431)	17.08 (343)	21.56 (433)	18.58 (373)	3.14 (1.58)

*—Reversed individual scores used for the calculation of the Index of Unhealthy Food Choices.

**Table 3:** Univariate linear regression of the Index of Unhealthy Food Choices (IUFC) as the dependent variable.

Variable	Categories of Variable	B (Standard Error)	β	95% CI	*p*
eHL		−0.11 (0.04)	−0.07	−0.18–−0.04	0.003
FL		−0.16 (0.03)	−0.13	−0.21–−0.11	<0.001
HL	problematic *				
	inadequate	1.62 (0.63)	0.06	0.38–2.86	0.011
	sufficient	−0.45 (0.66)	−0.02	−1.74–0.85	0.499
	undetermined	−0.51 (0.47)	−0.03	−1.44–0.42	0.278
Eating motivations	health-related	−0.96 (0.07)	−0.31	−1.09–−0.83	<0.001
	emotional	0.42 (0.04)	0.22	0.34–0.51	<0.001
	economic	1.08 (0.08)	0.29	0.93–1.24	<0.001
	social	0.04 (0.09)	0.01	−0.14–0.23	0.641
	environmental	−0.46 (0.05)	−0.19	−0.57–−0.36	<0.001
	marketing	1.25 (0.07)	0.37	1.12–1.39	<0.001
Age		−0.21 (0.01)	−0.33	−0.24–−0.19	<0.001
Gender	female *				
	male	3.38 (0.37)	0.20	2.66–4.1	<0.001
Place of residence	rural *				
	urban below 20,000 inhabitants	0.66 (0.62)	0.03	−0.55–1.87	0.285
	urban 20,000–100,000 inhabitants	−0.37 (0.51)	−0.02	−1.37–0.63	0.470
	urban 100,000–200,000 inhabitants	0.38 (0.71)	0.01	−1.01–1.78	0.590
	urban 200,000–500,000 inhabitants	0.05 (0.70)	0.002	−1.31–1.42	0.937
	urban above 500,000 inhabitants	−0.82 (0.63)	−0.03	−2.05–0.4	0.188
Education	secondary *				
	lower than secondary	1.96 (0.53)	0.09	0.92–3.01	<0.001
	post-secondary non-university	1.06 (0.62)	0.04	−0.16–2.28	0.089
	university Bachelors	−0.17 (0.62)	−0.01	−1.37–1.04	0.787
	university Masters	−1.72 (0.50)	−0.09	−2.7–−0.73	0.001
Marital status	married *				
	in partnership	1.72 (0.54)	0.07	0.66–2.77	0.001
	single	2.03 (0.45)	0.11	1.15–2.91	<0.001
	widowed, divorced, or separated	−1.61 (0.62)	−0.06	−2.83–−0.38	0.010
Vocational status	employee *				
	self-employed or farmer	−1.7 (0.58)	−0.07	−2.83–−0.57	0.003
	retired or on disability pension	−3.8 (0.69)	−0.12	−5.16–−2.44	<0.001
	high school or university student	2.37 (0.70)	0.08	1.00–3.74	0.001
	vocationally passive incl. unemployed	−0.26 (0.50)	−0.01	−1.24–0.72	0.602
Monthly net income pers household member	1501–3000 PLN *				
	not more than 1500 PLN	0.04 (0.56)	0.002	−1.07–1.15	0.945
	3001–5000 PLN	−0.06 (0.51)	−0.003	−1.07–0.94	0.906
	more than 5000 PLN	0.11 (0.63)	0.004	−1.12–1.34	0.862
	refusal of response	−0.48 (0.58)	−0.02	−1.61–0.65	0.404
Number of people in a household	3 persons				
	1 person (only respondent) *	−1.85 (0.72)	−0.06	−3.26–−0.45	0.010
	2 persons	−1.31 (0.52)	−0.07	−2.34–−0.29	0.012
	4 persons	0.73 (0.51)	0.04	−0.27–1.74	0.154
	more than 4 persons	1.77 (0.58)	0.08	0.63–2.91	0.002
Involvement in purchases of consumed food	almost always *				
	sometimes or less often	1.43 (0.73)	0.05	−0.01–2.87	0.051
	often	2.11 (0.72)	0.07	0.69–3.52	0.004
	very often	1.39 (0.58)	0.06	0.24–2.53	0.018
	always	−0.20 (0.47)	−0.01	−1.12–0.71	0.660
Chronic disease	no *				
	yes	1.41 (0.59)	0.05	0.24–2.57	0.018
Self-assessment of health status	good *				
	unsatisfactory	−1.29 (0.76)	−0.04	−2.78–0.21	0.092
	satisfactory	−1.71 (0.51)	−0.08	−2.71–−0.72	0.001
	very good	0.18 (0.47)	0.01	−0.74–1.11	0.697
	perfect	1.27 (0.87)	0.03	−0.43–2.98	0.144
Accessing influencer’s web pages	no use *				
	1–3 times in the last month	0.30 (0.47)	0.02	−0.62–1.21	0.523
	once weekly	0.35 (0.58)	0.01	−0.78–1.47	0.548
	2–3 times a week	1.93 (0.62)	0.08	0.71–3.14	0.002
	at least 4 times a week	1.11 (0.74)	0.04	−0.33–2.56	0.131
Use of the Internet daily	2–3 h *				
	not more than 1 h	−1.37 (0.74)	−0.05	−2.82–0.07	0.063
	>1 to 2 h	−1.16 (0.57)	−0.06	−2.28–−0.05	0.040
	>3 to 4 h	0.10 (0.60)	0.00	−1.08–1.28	0.869
	>4 to 5 h	1.75 (0.67)	0.07	0.43–3.07	0.009
	>5 h	2.39 (0.57)	0.11	1.26–3.51	<0.001
Watching TV daily	>1 to 2 h *				
	no use	−0.22 (0.64)	−0.01	−1.49–1.04	0.728
	not more than 0.5 h	−1.97 (0.73)	−0.07	−3.40–−0.55	0.007
	>0.5 to 1 h	0.20 (0.61)	0.01	−1.00–1.41	0.743
	>2 to 3 h	0.85 (0.59)	0.04	−0.31–2.00	0.151
	>3 to 4 h	0.79 (0.70)	0.03	−0.59–2.18	0.260
	>4 h	1.54 (0.71)	0.06	0.16–2.93	0.029

Abbreviations: PLN—polish zloty, TV—television, HL—health literacy, FL—food literacy, eHL—e-health literacy, B—regression coefficient, β—standardized regression coefficient, 95% CI—95% confidence interval, *—reference category of the variable in the regression model.

**Table 4:** Multiple linear regression of the Index of Unhealthy Food Choices as dependent variable (ANOVA, F = 18.584, *p* < 0.001, R2 = 0.368).

Variable	Categories of Variable	B (Standard Error)	β	95% CI	*p*
eHL		0.05 (0.04)	0.03	−0.02–0.12	0.189
FL		0.005 (0.03)	0.004	−0.06–0.07	0.879
HL	problematic *				
	inadequate	−0.38 (0.55)	−0.01	−1.46–0.70	0.492
	sufficient	0.95 (0.58)	0.03	−0.18–2.08	0.099
	undetermined	−0.49 (0.43)	−0.02	−1.33–0.35	0.251
Eating motivations	health-related	−0.83 (0.07)	−0.27	−0.98–−0.69	<0.001
	emotional	0.22 (0.04)	0.12	0.14–0.30	<0.001
	economic	0.41 (0.08)	0.11	0.25–0.56	<0.001
	social	−0.03 (0.09)	−0.01	−0.20–0.14	0.728
	environmental	−0.02 (0.05)	−0.01	−0.13–0.08	0.665
	marketing	0.62 (0.08)	0.18	0.47–0.78	<0.001
Age		−0.18 (0.02)	−0.28	−0.22–−0.14	<0.001
Gender	female *				
	male	3.54 (0.36)	0.21	2.84–4.23	<0.001
Place of residence	rural *				
	urban below 20,000 inhabitants	0.36 (0.51)	0.01	−0.64–1.36	0.477
	urban 20,000–100,000 inhabitants	−0.1 (0.43)	0.00	−0.94–0.74	0.821
	urban 100,000–200,000 inhabitants	0.55 (0.60)	0.02	−0.62–1.72	0.353
	urban 200,000–500,000 inhabitants	−0.13 (0.58)	0.00	−1.28–1.01	0.820
	urban above 500,000 inhabitants	−0.48 (0.53)	−0.02	−1.53–0.56	0.364
Education	secondary *				
	lower than secondary	0.25 (0.46)	0.01	−0.64–1.15	0.577
	post-secondary non-university	0.63 (0.51)	0.02	−0.38–1.64	0.224
	university Bachelors	−0.53 (0.51)	−0.02	−1.54–0.48	0.300
	university Masters	−0.20 (0.43)	−0.01	−1.06–0.65	0.639
Marital status	married *				
	in partnership	0.32 (0.48)	0.01	−0.62–1.27	0.504
	single	−1.17 (0.47)	−0.06	−2.08–−0.25	0.012
	widowed, divorced, or separated	−0.33 (0.55)	−0.01	−1.41–0.75	0.546
Vocational status	employee *				
	self-employed or farmer	−0.33 (0.49)	−0.01	−1.29–0.63	0.500
	retired or on disability pension	0.78 (0.65)	0.03	−0.49–2.04	0.228
	high school or university student	−1.26 (0.69)	−0.04	−2.61–0.09	0.068
	vocationally passive incl. unemployed	−0.46 (0.45)	−0.02	−1.36–0.43	0.308
Monthly net income per household member	1501–3000 PLN *				
	not more than 1500 PLN	−0.32 (0.48)	−0.01	−1.26–0.63	0.511
	3001–5000 PLN	0.03 (0.43)	0.00	−0.81–0.87	0.951
	more than 5000 PLN	−0.05 (0.53)	0.00	−1.09–0.99	0.930
	refusal of response	−0.96 (0.49)	−0.04	−1.92–0.01	0.051
Number of people in a household	3 persons				
	1 person (only respondent) *	−1.69 (0.70)	−0.06	−3.05–−0.32	0.016
	2 persons	−0.64 (0.48)	−0.03	−1.58–0.30	0.180
	4 persons	−0.45 (0.43)	−0.02	−1.29–0.39	0.295
	more than 4 persons	0.53 (0.50)	0.02	−0.44–1.51	0.285
Involvement in purchases of consumed food	almost always *				
	sometimes or less often	−1.44 (0.64)	−0.05	−2.69–−0.18	0.025
	often	−0.33 (0.61)	−0.01	−1.53–0.87	0.588
	very often	−0.10 (0.49)	0.00	−1.05–0.85	0.839
	always	−0.49 (0.4)	−0.03	−1.28–0.29	0.219
Chronic disease	no *				
	yes	−0.30 (0.51)	−0.01	−1.3–0.7	0.553
Self-assessment of health status	good *				
	unsatisfactory	−0.88 (0.71)	−0.03	−2.26–0.5	0.213
	satisfactory	−0.99 (0.50)	−0.05	−1.98–0	0.050
	very good	−0.08 (0.40)	0.00	−0.87–0.71	0.845
	perfect	0.55 (0.76)	0.01	−0.94–2.04	0.467
Accessing influencer’s web pages	no use *				
	1–3 times in the last month	0.19 (0.41)	0.01	−0.62–1.00	0.645
	once weekly	−0.66 (0.51)	−0.03	−1.65–0.34	0.194
	2–3 times a week	0.08 (0.54)	0.00	−0.98–1.15	0.878
	at least 4 times a week	−0.16 (0.64)	−0.01	−1.42–1.1	0.802
Use of the Internet daily	2–3 h *				
	not more than 1 h	−0.18 (0.62)	−0.01	−1.40–1.03	0.769
	>1 to 2 h	0.2 (0.47)	0.01	−0.72–1.13	0.668
	>3 to 4 h	−0.24 (0.51)	−0.01	−1.25–0.77	0.637
	>4 to 5 h	1.09 (0.58)	0.04	−0.04–2.22	0.059
	>5 h	1.30 (0.51)	0.06	0.30–2.30	0.011
Watching TV daily	>1 to 2 h *				
	no use	−1.10 (0.56)	−0.05	−2.19–−0.01	0.049
	not more than 0.5 h	−2.15 (0.61)	−0.07	−3.35–−0.96	<0.001
	>0.5 to 1 h	−0.25 (0.51)	−0.01	−1.24–0.74	0.618
	>2 to 3 h	0.86 (0.49)	0.04	−0.10–1.81	0.079
	>3 to 4 h	0.35 (0.59)	0.01	−0.80–1.50	0.554
	>4 h	0.58 (0.61)	0.02	−0.63–1.78	0.349

Abbreviations: PLN—polish zloty, TV—television, HL—health literacy, FL—food literacy, eHL—e-health literacy, B—regression coefficient, β—standardized regression coefficient, 95% CI—95% confidence interval, *—reference category of the variable in the regression model.

## Data Availability

The data are not publicly available due to privacy and ethical restrictions. The authors did not include in the information about the study provided to the participants that public access to the data obtained during the survey may be considered. Access to the data will be granted on a case-by-case basis to a justified request after receiving consent from the Bioethical Committee at Jagiellonian University.
